# Identification and Characterization of *Hibiscus mutabilis* Varieties Resistant to *Bemisia tabaci* and Their Resistance Mechanisms

**DOI:** 10.3390/insects15060454

**Published:** 2024-06-14

**Authors:** Juan Wei, Xiaoli Liu, Chan Li, Yuanzhao Yang, Cancan Song, Yihao Chen, Qiongda Ciren, Chunxian Jiang, Qing Li

**Affiliations:** 1 College of Agronomy, Sichuan Agricultural University, Chengdu 611130, China; 18352887796@163.com (J.W.); songcan1993@163.com (C.S.);; 2 Chengdu Botanical Garden (Chengdu Park Urban Plant Science Research Institute), Chengdu 610083, China; liuzj010814@163.com (X.L.);

**Keywords:** resistance screening, herbivore-induced plant volatiles, metabolite, metabolic pathway

## Abstract

**Simple Summary:**

This study investigated the resistance of *Hibiscus mutabilis* varieties to the whitefly *Bemisia tabaci*. It identified the highly resistant variety Jinqiusong (JQS) and moderately resistant Bairihuacai (BRHC), which showed significantly lower *B. tabaci* populations than the susceptible variety Chongbanbai (CBB). Metabolomic analysis revealed that fifteen key metabolites are linked to resistance, with the phenylpropanoid biosynthesis pathway being critical in defense. Additionally, volatile compounds emitted by the resistant varieties deterred the pest’s behavior. The findings offer a theoretical basis for breeding insect-resistant *H. mutabilis* varieties, providing a sustainable strategy for managing *B. tabaci* infestations.

**Abstract:**

*Hibiscus mutabilis*, the city flower of Chengdu, is culturally significant and has nutritional and medicinal benefits. However, frequent infestations of *Bemisia tabaci* have caused economic losses. This study aimed to identify insect-resistant *H. mutabilis* varieties. Over two years, varieties like Jinqiusong, Zuiyun, and Zuifurong showed moderate to high resistance based on reproductive indices. Assessments of antixenosis and developmental impacts revealed that adult *B. tabaci* exhibited low selectivity toward these resistant varieties, indicating a strong repellent effect. Gas chromatography-mass spectrometry analysis identified volatile organic compounds, such as alcohols, alkanes, and terpenes. Notably, 2-ethylhexanol and 6-methylheptanol exhibited repellent properties. Using nontargeted metabolomics, this study compared the metabolite profiles of the insect-resistant variety Jinqiusong (JQS), moderately resistant Bairihuacai (BRHC), and highly susceptible Chongbanbai (CBB) post *B. tabaci* infestation. Fifteen key metabolites were linked to resistance, emphasizing the phenylpropanoid biosynthesis pathway as crucial in defense. These findings offer a theoretical foundation for breeding insect-resistant *H. mutabilis* varieties and developing eco-friendly strategies against *B. tabaci* infestations.

## 1. Introduction

*Hibiscus mutabilis*, a member of the Malvaceae family, is an ornamental plant indigenous to Hunan Province, China, with a cultivation history spanning nearly 3000 years. The plant is now cultivated in Japan, Korea, India, Cuba, Thailand, Vietnam, and Bangladesh. Since 1983, *H. mutabilis* has been designated as the city flower of Chengdu, Sichuan Province, earning Chengdu the nickname “Hibiscus City” [1]. This species not only holds significant cultural heritage but is also recognized for its nutritional and medicinal properties, qualifying it as a Chinese medicinal and food substance. The historical and medicinal uses of *H. mutabilis* are particularly relevant to the study of pest resistance, as they provide insights into the plant’s resilience and potential mechanisms of defense that can be explored and applied in pest management strategies. Classical texts such as the *Illustrated Classic of Materia Medica* and the *Compendium of Materia Medica* describe the flowers and leaves of *H. mutabilis* as “spicy and nontoxic” with benefits including cooling the blood, detoxification, reducing swelling, and pain relief. Recent publications, including *Edible Medicinal and Nonmedicinal Plants* (2014) and the 2015 edition of the *Chinese Pharmacopoeia*, have documented *H. mutabilis* as a new medicinal material [2]. Chemical analyses revealed that *H. mutabilis* contains steroids, hibiscins, tannins, carbohydrates, and flavonoids and is particularly rich in phenolics, thus demonstrating strong antioxidant properties [3,4,5]. *H. mutabilis* extracts are extensively utilized in cosmetics, food ingredients, and additives. Moreover, the stem bark is used in paper manufacturing, while the flowers are employed in various culinary dishes [6,7,8,9]. Thus, *H. mutabilis* represents a multifunctional crop with ornamental, medicinal, edible, textile, and papermaking applications, underscoring its considerable research value.

The whitefly, *Bemisia tabaci* (Gennadius, *B. tabaci*), from the Aleyrodidae family within the order Hemiptera, is one of the most damaging invasive species in the world. This pest infests over 600 plant species, causing substantial agricultural economic losses [10,11]. The Food and Agriculture Organization of the United Nations has identified *B. tabaci* as the second most significant plant pest in the world due to the rapid dissemination, broad host range, and severe economic impacts [12]. First recognized in Greece in 1889, *B. tabaci* was reported in China by 1949, initially with a limited population. Following the introduction of *Euphorbia pulcherrima* from overseas in 1994, *B. tabaci* emerged as a major agricultural pest in China, as the plant serves as a favorable host, supporting higher reproduction rates and population growth of *B. tabaci* [13,14,15,16]. The burgeoning *H. mutabilis* industry has experienced increased demand in recent years [17]. Nevertheless, the frequent outbreaks of *B. tabaci* pose significant challenges. *B. tabaci* compromises plant health by stunting growth and inducing leaf yellowing through sap extraction. *B. tabaci* also secretes honeydew, leading to sooty mold that further hinders plant growth. Moreover, the rapid development of *B. tabaci* insecticide resistance and its adverse effects on natural predators complicate control efforts, severely restricting *H. mutabilis* yields and exacerbating economic losses [18,19,20,21].

This study aimed to identify *H. mutabilis* varieties with inherent resistance to *B. tabaci* through comparative analysis of reproductive indices, antixenosis, and antibiosis tests. Additionally, gas chromatography-mass spectrometry (GC-MS) analysis was employed to determine the differences in volatile compounds between *B. tabaci*-resistant and *B. tabaci*-susceptible *H. mutabilis* leaves. Combined with olfactory response tests on *B. tabaci*, this approach identified specific *H. mutabilis* volatiles that attract or repel the pest. Nontargeted metabolomics technology was applied to investigate the metabolite variations in *H. mutabilis* following infestation, to elucidate the physiological and biochemical mechanisms underlying the resistance of different varieties.

This research aims to highlight key secondary metabolites and metabolic pathways in *H. mutabilis* to provide a theoretical foundation for selecting and breeding *B. tabaci*-resistant *H. mutabilis* varieties and supporting the development of sustainable pest management strategies.

## 2. Materials and Methods

### 2.1. Establishment of B. tabaci Population

The initial population of *B. tabaci* was sourced from the *H. mutabilis* nursery at Chengdu Botanical Garden, Sichuan, China. *B. tabaci* specimens were subsequently biotyped using mitochondrial DNA cytochrome oxidase I (mtDNA COI) gene sequencing, chosen for its high resolution and reliability in distinguishing between different biotypes, confirming their classification as type Q [15]. For experimental purposes, 1-year-old plants of *H. mutabilis* variety Chongbanbai (CBB) were potted and infested with *B. tabaci* on leaves within enclosures of 100-mesh nylon netting. These were then maintained for breeding in the Entomology Laboratory of the Department of Plant Protection at Sichuan Agricultural University. The breeding was continued for over three generations until the whiteflies matured into adults. Environmental conditions were controlled at a temperature of 25 °C ± 1 °C, humidity ranging from 40% to 50%, and a photoperiod of L:D = 16:8 h. These conditions were chosen to closely mimic the natural habitat of *B. tabaci*, ensuring optimal growth and reproduction. The temperature of 25 °C ± 1 °C provides a stable environment conducive to the development of *B. tabaci*, while the humidity range of 40% to 50% prevents desiccation without promoting mold growth. The photoperiod of L = 16:8 h simulates natural daylight cycles, promoting normal behavioral and physiological responses in *B. tabaci*.

### 2.2. Plant Culture

**Figure 1:**
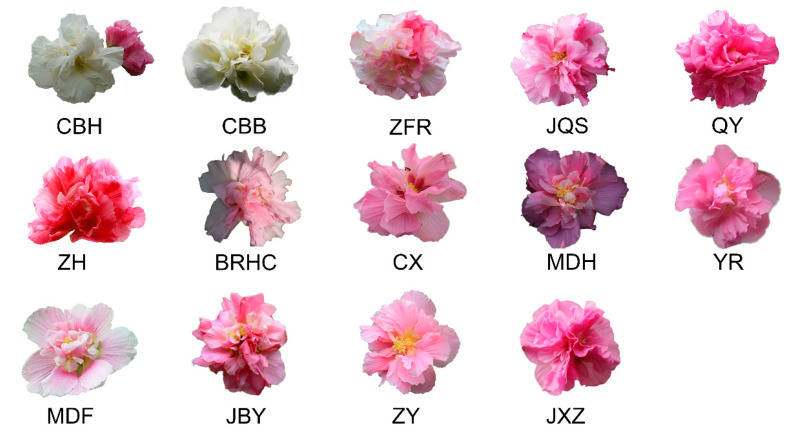
Fourteen varieties of *H. mutabilis*.

This study used 14 varieties of *H. mutabilis*, specifically Chongbanbaibianhong (CBH), CBB, Zuifurong (ZFR), Jinqiusong (JQS), Qiuyun (QY), Zuihong (ZH), Bairihuacai (BRHC), Caixia (CX), Mudanhong (MDH), Yurui (YR), Mudanfen (MDF), Jinbiyu (JBY), Zuiyun (ZY), and Jinxiuzi (JXZ) (Figure 1). Each variety was chosen for its diverse genetic background and potential differences in resistance to *B. tabaci*. This diversity allowed for a comprehensive analysis of resistance mechanisms. All plant varieties were procured from the Chengdu Botanical Garden, Sichuan, China (104.14° E, 30.76° N). In the late spring and early summer of 2021, vigorous and healthy disease-free 1-year-old cuttings of *H. mutabilis* were transplanted into round plastic pots, 15 cm in diameter and approximately 20 cm in height, where each pot contained one plant. Plants were grown in Yuexi grass charcoal soil and received balanced fertilizer and water management. During nonexperimental periods, plants were enclosed in cages with 100-mesh screens to prevent pest infestation.

### 2.3. Field Resistance Evaluation

In June 2021, the resistance of the hibiscus seedlings to *B. tabaci* was evaluated in the nursery of Chengdu Botanical Garden using a completely randomized block design with 14 treatments, each replicated five times. Healthy 1-year-old, potted hibiscus seedlings, free of pests and diseases, were selected. One plant per pot was placed within a 100-mesh light nylon mesh trellis (4 × 2.2 × 1.6 m), maintaining a 20 cm distance between pots. Approximately 500 laboratory-reared *B. tabaci* adults were released into the enclosure, allowing them to disperse freely. The number of *B. tabaci* adults was recorded at 24, 48, and 72 h intervals. Leaves from the upper, middle, and lower sections were inspected for *B. tabaci* adults, with counts conducted on the undersides of leaves. One-week pos tinfestation, three leaves per plant were collected, stored in plastic bags, and used for egg counting under a stereo microscope (40× magnification). Varieties showing differential resistance were selected for subsequent resistance mechanism studies. A reselection test was performed in May 2022 using identical methods and under identical environmental conditions.

Resistance was assessed using the reproduction index described by Taylor et al. [22] and according to resistance evaluation criteria established by Millan et al. [23]. The methodology and framework used in this study align with the guidelines discussed by Stenberg and Muola [24]. CBB, a susceptible variety, served as the control. The reproduction index, calculated as the ratio of the number of eggs laid on the test variety to the number laid on the control variety (CBB), provided resistance grades. This metric quantified the reproductive success of *B. tabaci*, allowing us to identify hibiscus varieties with higher resistance.

Reproduction index = 0 (immunity);

Reproduction index = 1–20 (resistant);

Reproduction index = 21–50 (moderately resistant);

Reproduction index = 51–99 (sensitive);

Reproduction index ≥ 100 (highly susceptible).

### 2.4. Antixenosis and Antibiosis Experiments of H. mutabilis to B. tabaci

Following 2 years of field resistance screening, seven *H. mutabilis* varieties (CBB, BRHC, JQS, ZFR, MDH, ZY, and JXZ) exhibiting differential resistance were selected for antixenosis (measuring how much damage or how many herbivores a plant attracts) and antibiosis (assessing how suitable the plant is for the herbivore) tests [24]. Each variety was represented by one potted seedling, placed in a circle within a 100-mesh nylon mesh trellis (2.5 m × 2.5 m × 1.6 m). Approximately 300 *B. tabaci* adults were released at the center of the circle, 50 cm from the seedlings, allowing free movement and host selection. *B. tabaci* infestation on each variety was recorded at 24, 48, and 72 h. The experiment was repeated five times to calculate the selection rate of *B. tabaci* on different *H. mutabilis* varieties. The selection rate refers to the ratio of the number of *B. tabaci* on a particular plant variety to the total number of *B. tabaci* in the experiment. This rate indicates the attractiveness of each variety to *B. tabaci* and is significant for understanding plant resistance mechanisms.

Antibiosis experiments were conducted at 25 °C ± 1 °C. Uniformly grown *H. mutabilis* seedlings were infested, and insects were allowed to lay eggs freely. Adults were removed 12 h post infestation, and egg counts were conducted. Following the method of Novaes et al. [25], leaves with uniformly distributed eggs were selected, retaining 30–50 eggs per leaf as one replicate. Excess eggs were gently removed with a brush, and eggs were monitored post-hatching every 24 h until adult emergence. The developmental duration and survival rate of *B. tabaci* at each insect stage on different *H. mutabilis* varieties were calculated, with three replicates of the experiment conducted.

### 2.5. Determination of HIPVs Components in Different Resistant H. mutabilis Varieties and Olfactory Behavioral Responses of B. tabaci

#### 2.5.1. Collection and Analysis of Volatiles from *H. mutabilis* Leaves

Healthy plants from the insect-resistant variety JQS and the insect-susceptible variety CBB were inoculated with *B. tabaci*. Adults from a 2 h starvation treatment were confined to upper, middle, and lower leaves using a 100-mesh nylon mesh cover (20 × 20 cm), with each leaf hosting 30 individuals. After 4 days of feeding, adults and eggs were removed, and leaves were rinsed with pure water to collect HIPVs. Volatiles from *H. mutabilis* leaves were collected using headspace adsorption solid-phase microextraction (Supplementary Material Figure S1) and categorized into four treatments (healthy insect-resistant, healthy insect-susceptible, pest-infested insect-resistant, and pest-infested insect-susceptible plants), adding 6-methyl-5-hepten-2-one as an internal standard, with three replicates per treatment. Volatiles were analyzed using Shimadzu gas chromatography-mass spectrometry (GCMS-TQ8040). Chromatographic conditions were set at 40 °C for 3 min, increased to 160 °C at 5 °C/min, then to 260 °C at 10 °C/min, and held for 5 min. Mass spectrometry conditions included a GC-MS interface temperature of 280 °C, no shunt ratio, an EI ion source at 70 eV, and a mass range from 35 to 500 amu in the full-scan mode, utilizing a NIST 11 search library.

#### 2.5.2. Determination of Olfactory Behavioral Responses in *B. tabaci*

Building on the identified volatile compounds from *H. mutabilis* leaves, eight representative compounds were selected for *B. tabaci* olfactory behavioral tests (Supplementary Material Table S1). Solutions of these compounds were prepared in liquid paraffin at 10^−3^, 10^−2^, and 10^−1^ mg/mL. A four-arm olfactometer, as described by Hou et al. [26] and Mizuno et al. [27], was used to assess the selection rate of *B. tabaci* for specific compounds at varying concentrations, with liquid paraffin serving as a control. The airflow of the olfactometer was set at 500 mL/min to channel odors towards the insects. Fifty adult *B. tabaci* were placed in the center port, and the test duration was 30 min. The number of insects choosing each odor source was recorded, with each treatment replicated four times. The formulas were applied to calculate the reaction rate, selectivity, and selection coefficient [28].

A selection coefficient > 0 indicates attraction to the odor source, with higher values indicating stronger attraction. Conversely, a selection coefficient < 0 indicates repellence by the odor source.

### 2.6. Metabolites and Metabolic Pathway Involved in B. tabaci Resistance and Susceptibility

The susceptible variety CBB, the moderately resistant variety BRHC, and the resistant variety JQS were evaluated in two treatments: healthy plants and insect-infested plants. Twenty adult *B. tabaci* were placed on the third leaf from each healthy *H. mutabilis* plant after a 2 h starvation treatment. After 4 days of feeding, insects were removed, and leaves were rinsed and harvested 6 h later to capture immediate metabolic responses to infestation. This time point is critical for observing early defense mechanisms and rapid changes in metabolite levels, which are essential for activating downstream defense pathways and understanding plant-insect interactions. Corresponding tissue samples from unaffected healthy plants were also collected as controls. Each type of sample was collected with six biological replicates, and tissues were immediately frozen in liquid nitrogen and stored at −80 °C. Metabolomic sample preparation was conducted as described by Vasilev et al. [29]. The metabolome analysis was performed by Panomix Co. Ltd. (Suzhou, China). Specific metabolite analysis is essential for understanding resistance mechanisms, as metabolites are key to plant defense. By comparing the metabolite profiles of susceptible, moderately resistant, and resistant varieties under healthy and infested conditions, we can identify compounds associated with resistance to *B. tabaci*. These may include secondary metabolites, signaling molecules, or other chemicals that enhance the plant’s defense against *B. tabaci* attacks.

### 2.7. Data Analysis

Metabolites data preprocessing involved converting raw mass spectra files into the mzXML format using the MS Convert tool from the ProteoWizard software package (v 3.0. 8789). Peak detection, filtering, and alignment were conducted using the R XCMS package. Multivariate statistical analysis of the sample data was performed using the R package Ropls. Important variables in the projection were identified using the variable importance in projection (VIP) score from the OPLS-DA model, with a threshold value > 1 and combined with *p*-values from Student’s *t*-test (*p* < 0.05) to identify differentially expressed metabolites. The choice of these statistical methods and software was based on their robustness and wide acceptance in metabolomics data analysis, ensuring accurate and reliable identification of significant metabolites.

Statistical analysis of the experimental data was performed using Excel 2010 and SPSS 20.0. Differences among treatments were analyzed using one-way analysis of variance followed by Duncan’s multiple range test (*p* < 0.05). These methods were chosen for their ability to accurately compare multiple groups and identify significant differences. Graphs were generated using GraphPad Prism 7.

## 3. Results

### 3.1. Comparison of Resistance to B. tabaci in Different H. mutabilis Varieties

Over 2 years of surveys, we observed that the CBB variety exhibited the highest egg-laying by *B. tabaci* in both 2021 and 2022, demonstrating stable susceptibility traits. Therefore, CBB was employed as the susceptible control variety due to its representative nature. In 2021, significant differences were noted in the number of adult *B. tabaci* and eggs laid on different *H. mutabilis* varieties (*p* < 0.05). Among the 14 *H. mutabilis* varieties, the highest 3-day average number of adults was recorded on the JXZ variety at 5.27 heads per leaf, which was significantly higher than all other varieties except CBB (*p* < 0.05), which had 5.07 heads per leaf. Varieties such as ZFR (1.51 heads heads/leaf), ZY (2.02 heads/leaf), and JQS (2.13 heads/leaf) had significantly lower 3 d average adult counts than the other varieties (*p* < 0.05). For egg production, CBB had the highest average at 29.93 grains per leaf, which was significantly more than that in the other varieties except for YR (26.87 grains/leaf) (*p* < 0.05). ZY had the lowest mean egg production at four grains/leaf (Supplementary Material Table S2). Similar trends continued into 2022, showing significant differences in the number of adults and eggs laid by *B. tabaci* on different *H. mutabilis* varieties (*p* < 0.05). CBB had the highest 3-day average number of adults (6.73 heads/leaf) and egg production (21.47 grains/leaf). Conversely, varieties like JQS (4.13 grains/leaf) and MDH (6.47 grains/leaf) recorded significantly lower egg production than CBH (17.60 grains/leaf) and CBB, with minor differences from other varieties (Supplementary Material Table S3).

### 3.2. Effects of Different H. mutabilis Varieties on Antixenosis of B. tabaci

**Figure 2:**
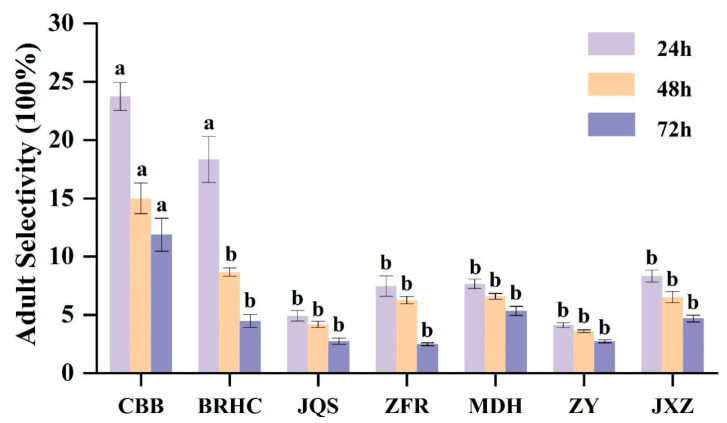
The repellent tendency of different *H. mutabilis* cultivars to *B. tabaci* adults. The letters in the graph indicate the significance of the difference between the different varieties at the same time (one-way ANOVA, *p* < 0.05). Error bars represent the standard error.

Based on the results from 2 years of field resistance screening, seven varieties (CBB, BRHC, JQS, ZFR, MDH, ZY, and JXZ) with differing resistance levels were selected for further evaluation of antixenosis and antibiosis. The preference of adult *B. tabaci* for these varieties showed significant differences (*p* < 0.05) at various time points (24, 48, and 72 h). Specifically, after 24 h, CBB exhibited the highest selection rate (23.73%), which significantly surpassed those of all other varieties except for BRHC (18.33%). This trend continued at 48 and 72 h, with preferences in descending order being CBB (11.87%), MDH (5.33%), JXZ (4.67%), BRHC (4.47%), and then JQS and ZY (2.73%), followed by ZFR (2.47%). Thus, CBB may have a weak antixenosis effect on adult *B. tabaci*, whereas ZFR, ZY, and JQS may exhibit stronger antixenosis effects (Figure 2).

### 3.3. Effects of Different H. mutabilis Varieties on the Growth and Development of B. tabaci

**Figure 3:**
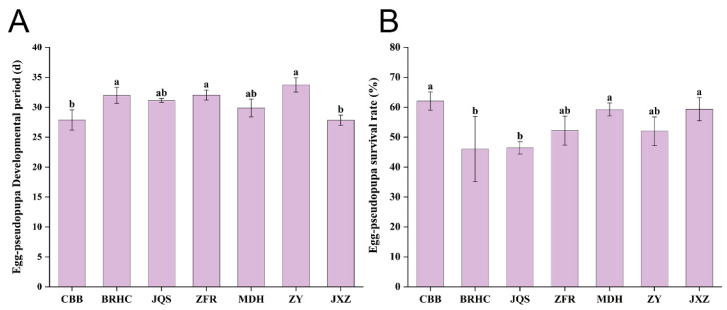
Impact of *H. mutabilis* varieties on *B. tabaci* development and survival. Comparative analysis of (**A**) the developmental period from egg to pseudopupa, and (**B**) the survival rate of *Bemisia tabaci* (egg to pseudopupa stage) across seven *H. mutabilis* varieties. Bars labeled with different letters indicate a statistically significant difference in the respective developmental and survival metrics among the tested *H. mutabilis* varieties (one-way ANOVA, *p* < 0.05). Error bars represent standard error.

*B. tabaci* exhibited significantly different developmental periods across the seven *H. mutabilis* varieties with varying levels of resistance and susceptibility. *B. tabaci* on the ZY variety recorded the longest developmental period at 33.75 days, which was significantly longer than that on JXZ and CBB (*p* < 0.05), although not significantly different from other varieties (*p* > 0.05). Conversely, JXZ showed the shortest growth cycle at only 27.85 days, which significantly differed from those of ZY, BRHC, and ZFR (*p* < 0.05) but not from those of the other varieties (*p* > 0.05). The growth cycle of *B. tabaci* from the longest to shortest on *H. mutabilis* varieties was in the order of ZY, ZFR, BRHC, JQS, MDH, CBB, and JXZ. Therefore, ZFR, BRHC, and ZY are relatively unsuitable for *B. tabaci* development. These findings suggest that different *H. mutabilis* varieties significantly affect the growth and development of *B. tabaci*, with preferred varieties facilitating shorter developmental periods and less hospitable varieties supporting longer periods (Figure 3A and Supplementary Material Table S4).

The survival rate of *B. tabaci* from egg to pseudopupa was highest on CBB at 62.08% and was significantly higher than that on BRHC (46.02%) and JQS (46.43%). The survival rates of first-instar larvae, third-instar larvae, pseudonymphs, and eggs did not significantly differ among the seven varieties. However, the survival rate of second-instar larvae was lowest on BRHC, which was significantly lower than other varieties (except for JQS). This indicates that JQS and BRHC are unsuitable for *B. tabaci* (Figure 3B and Supplementary Material Table S5).

### 3.4. Identification of HIPVs in Four H. mutabilis Samples

HIPVs from healthy and *B. tabaci*-infested CBB (insect-susceptible) and JQS (insect-resistant) *H. mutabilis* plants were collected via solid-phase microextraction and analyzed using GC-MS. A total of 40 volatile components were identified, comprising alkanes (12 compounds, 30%), alcohols (9 compounds, 22.5%), terpenes (7 compounds, 17.5%), ketones (3 compounds, 7.5%), esters (3 compounds, 7.5%), and benzene derivatives, aldehydes, and acids (3 compounds each, 5%) (Table 1). The analysis identified 24 metabolites in healthy CBB (CBB-H), 23 in healthy JQS (JQS-H), and 28 in both pest—induced CBB (CBB-I) and JQS (JQS-I).

**Figure 4:**
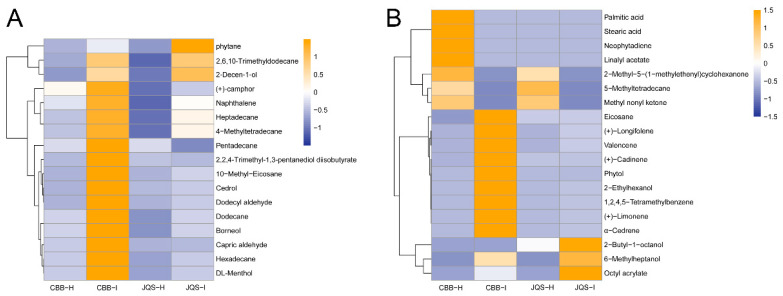
Comparative volatile metabolite profiles in healthy (H) or infested (I) *H. mutabilis* leaves under *B. tabaci* infestation. (**A**) highlights 17 metabolites present in both resistant (JQS) and susceptible (CBB) varieties, (**B**) contrasts 19 distinct metabolites in the two plant types. Color intensity reflects the volatile metabolite concentration-blue for lower, orange for higher.

These groups shared 17 volatiles, including dodecane, heptadecane, and hexadecane. The relative contents of these shared volatiles were calculated using the internal standard method, which showed that the volatile contents in pest-treated plants were higher than those in healthy plants, particularly in the insect-susceptible varieties. The results showed a general increase in the volatile content of pest-treated plants compared with that in healthy *H. mutabilis* plants, and a significant increase in the release of compounds such as (+)-camphor. The contents of pentadecane and 2,2,4-trimethyl-1,3-pentanediol diisobutyrate were decreased while those of the remaining shared constituents were increased in insect-infested leaves compared with those in healthy ones, particularly in the insect-resistant JQS variety (Figure 4A). These results suggest that *B. tabaci* feeding influences the release of volatiles from *H. mutabilis* leaves, which is potentially linked to the plant resistance mechanisms against *B. tabaci*.

We also found significant differences in the volatile components between pest-infested and healthy *H. mutabilis*. By analyzing the leaves of *H. mutabilis* with varying resistance to *B. tabaci* feeding before and after infestation, we identified 19 different components. These included 5-methyl-tetradecane, eicosane, 2-butyl-1-octanol, 2-ethylhexanol, 6-methylheptanol, phytol, and phytoene, among others. The types and contents of terpenoids were higher in the volatiles of *H. mutabilis* leaves after feeding by *B. tabaci* than those of healthy plants, indicating that terpenoids were the main components of volatiles in *B. tabaci*-induced *H. mutabilis* leaves. Infested plants newly produced substances such as (+)-limonene, (+)-caryophyllene, and 5-methyltetradecane while others were detected only in healthy plants, probably because *B. tabaci* infestation suppressed the host-defense response. In healthy *H. mutabilis*, substances such as neophytadiene appeared only in the highly susceptible variety CBB, whereas substances such as eicosanoids were detected in the insect-resistant variety JQS, which may be related to the insect resistance of *H. mutabilis* (Figure 4B).

### 3.5. Olfactory Behavioral Responses of B. tabaci to Different Concentrations of Compounds

Based on the volatile content from resistant and susceptible *H. mutabilis* leaves, eight representative single compounds were selected to evaluate their impact on the olfactory behavior of *B. tabaci*. The results indicated that dodecanal, cedrol, and α-cedrene significantly attracted *B. tabaci*, whereas stearic acid, 2-ethylhexanol, and 2-butyl-1-octanol demonstrated repellent effects. Conversely, eicosanoids and linalyl acetate did not significantly influence *B. tabaci* behavior. Notably, both attraction and repellence varied with the concentration of the compounds, with effects significantly more pronounced at optimal concentrations than those in the control group (Supplementary Material Table S6).

### 3.6. Nontargeted Metabolomics Reveal the Resistance Mechanisms of H. mutabilis against B. tabaci

#### 3.6.1. Quality Control Analysis of Metabolomic Samples

**Figure 5:**
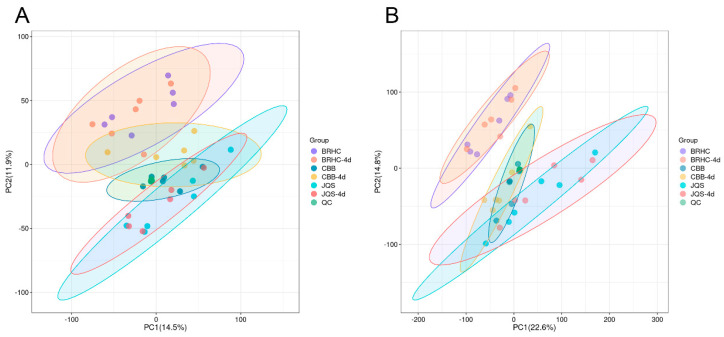
Principal component analysis (PCA) of volatile compound profiles in *H. mutabilis* smples. Scatter plots representing PCA scores of *H. mutabilis* samples analyzed in both (**A**) positive and (**B**) negative ion modes. Principal Component 1 (PC1) and Principal Component 2 (PC2) are plotted along the *x*-axis and *y*-axis, respectively, explaining 14.5% and 11.9% of the variance in A, and 22.6% and 14.8% in B. Each point corresponds to an individual sample, with distinct colors signifying different experimental groups. Ellipses denote the 95% confidence intervals for the group clusters, illustrating the variability and grouping within the data set.

The analysis included leaf metabolites and metabolic pathway changes in the susceptible variety CBB, the moderately resistant variety BRHC, and the resistant variety JQS after 4 days post infestation. A total of 608 metabolites were identified, with 433 species detected in the positive-ion mode and 175 in the negative-ion mode. Principle component analysis of samples within each group showed high-quality control aggregation, indicating good repeatability of the quality control and reliability of the test results. High reproducibility was also observed within each group, suggesting that the sample data were stable, reliable, and suitable for subsequent analyses (Figure 5).

#### 3.6.2. Differential Metabolite Analysis of Resistant and Susceptible *H. mutabilis* Materials under *B. tabaci* Infestation 

**Figure 6:**
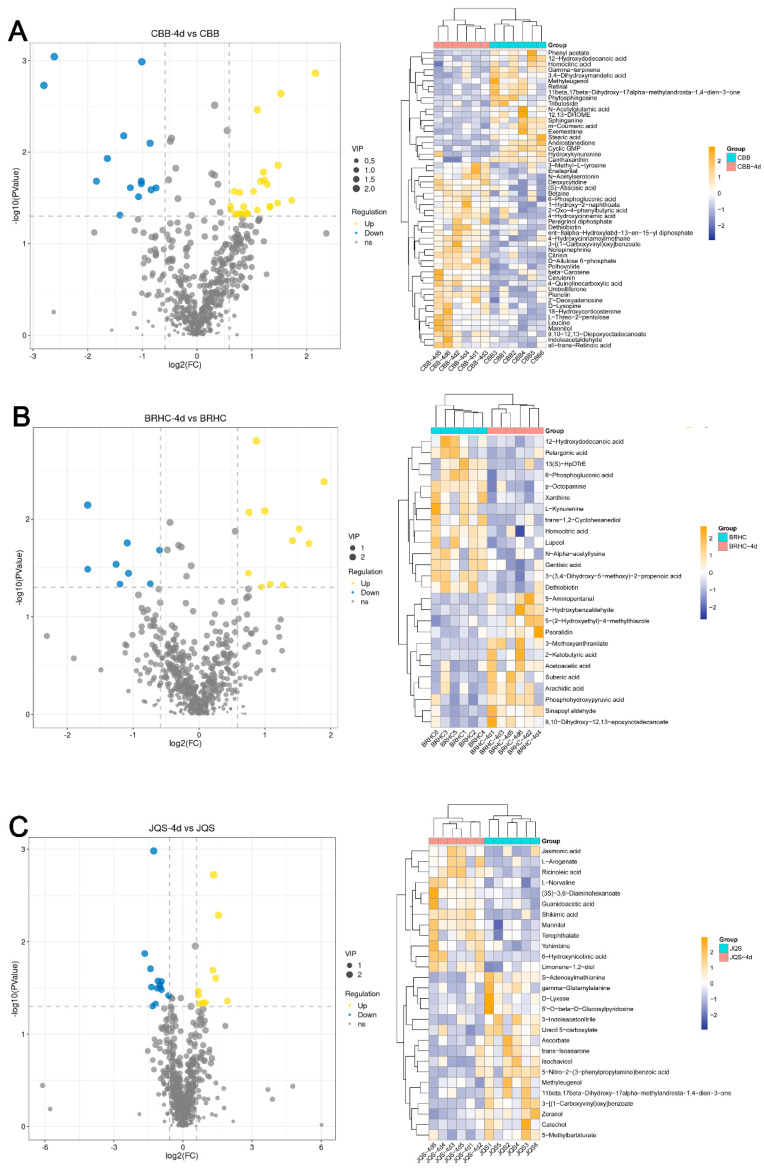
Differential metabolite expression in *H. mutabilis* in response to *B. tabaci* infestation. Volcano plots and hierarchical clustering heat maps illustrate the differential expression profiles of metabolites in *H. mutabilis* varieties and materials, respectively, in response to 4 days of *B. tabaci* infestation: the differential expression between the control and post-infestation (**A**) for the CBB variety, (**B**) for the JQS variety, and (**C**) for the BRHC variety. Left: volcano plots detail metabolite changes with log2 fold change (*x*-axis) and the −log10 *p*-value (*y*-axis), highlighting upregulated (in yellow) and downregulated (in blue) metabolites exceeding two-fold change with a *p*-value below 0.05, marked by dashed lines for change and significance thresholds. Right: heat maps show metabolite expression levels, from low (blue) to high (yellow), and feature dendrograms for clustering analysis of sample groups and metabolites.

Differential metabolites were screened with criteria set at fold change (FC) > 2 and VIP > 1. In total, 608 metabolites were detected, of which 219 were known. Analysis revealed 53 differential metabolites between CBB and CBB at 4 days post infestation (CBB-4d). A total of 33 metabolites were upregulated in CBB-4d, including betaine, leucine, indoleacetaldehyde, and 4-hydroxycinnamoylmethane, whereas 20 metabolites were downregulated, such as gamma-terpinene, m-coumaric acid, methyleugenol, and N-acetylglutamic acid (Figure 6A). In BRHC, 26 differential metabolites were identified between the untreated and 4-day post infestation samples. Twelve metabolites, including 2-ketobutyric acid and acetoacetic acid, were upregulated in BRHC-4d, whereas 14, including *p*-octopamine and xanthine, were downregulated (Figure 6B). JQS-4d showed 12 upregulated metabolites, including 6-hydroxynicotinic acid and mannitol, and several downregulated metabolites such as catechol and D-lyxose (Figure 6C). Of note, 3-[(1-carboxyvinyl) oxy] benzoate was upregulated in CBB following 4 days of feeding by *B. tabaci* but downregulated in JQS under similar conditions. Additionally, dethiobiotin exhibited upregulation in CBB and downregulation in BRHC. These differential expressions likely reflect inherent genetic variations in the defense mechanisms of *H. mutabilis* against *B. tabaci*. Mannitol was upregulated in both resistant and susceptible varieties, suggesting its potential as a candidate metabolite for resistance studies.

#### 3.6.3. Potential Biochemical Markers of *H. mutabilis* under *B. tabaci* Infestation

**Figure 7:**
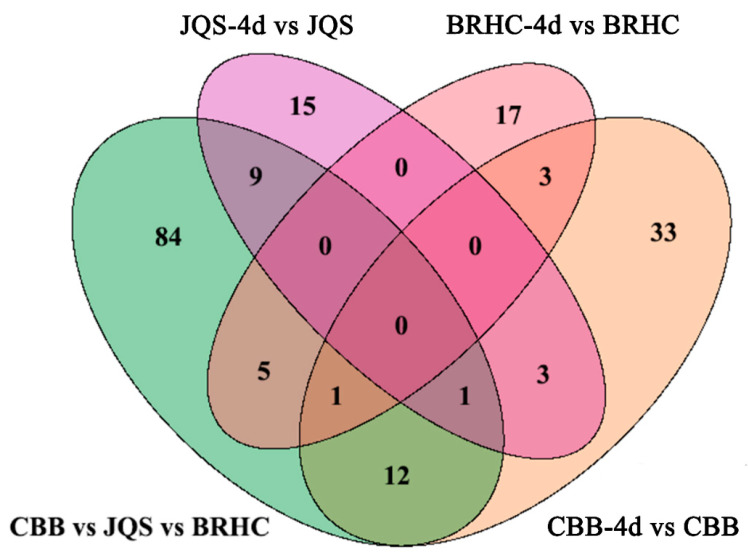
Comparative analysis of metabolite changes in *H. mutabilis* varieties following *B. tabaci* infestation. The Venn diagram depicts the comparative quantification of differential metabolites identified among the *H. mutabilis* varieties CBB, JQS, and BRHC when challenged with *B. tabaci* for 4 days. Each circle represents a comparison between the metabolite profiles pre-and post-infestation for a given variety: the green circle represents CBB vs. JQS vs. BRHC, the purple circle represents JQS-4d vs. JQS, and the pink circle represents BRHC-4d vs. BRHC. The orange circle represents CBB-4d vs. CBB. Overlapping regions indicate the number of metabolites that are differentially expressed in common between the different comparisons, while unique sections denote variety-specific changes.

For this analysis, only the 219 known metabolites were considered to identify potential biochemical markers in *H. mutabilis* and *B. tabaci* interactions. These metabolites were categorized based on their resistance properties (*n* = 15), moderate resistance properties (*n* = 17), susceptibility properties (*n* = 33), and host properties (*n* = 84) (Figure 7). Resistance and susceptibility traits were further analyzed for their potential as biochemical markers. In the resistant material, 15 metabolites demonstrated significant differences between induced and uninduced tissues, indicating their resistance traits [30]. Characteristic metabolites for resistance included catechol, isochavicol, 6-hydroxynicotinic acid, and 5-methylbarbiturate. Except for 6-hydroxynicotinic acid and limonene-1,2-diol, which were upregulated, all other characterized metabolites were predominantly downregulated. The downregulated metabolites generally exhibited more substantial changes than the upregulated ones (Table 2). In the susceptible variety, 33 metabolites displayed significant differences between induced and uninduced conditions and were classified as susceptible traits. Twenty metabolites, including betaine and indoleacetaldehyde, were upregulated while 13, including gamma-terpinene and m-coumaric acid, were downregulated. Notably, upregulated metabolites such as indole-acetaldehyde and 4-hydroxycinnamic acid exhibited higher log2FC values (*p* < 0.05), indicating more pronounced changes within susceptible materials (Supplementary Material Table S7).

#### 3.6.4. Differential Metabolite KEGG Enrichment Pathway Analysis

**Figure 8:**
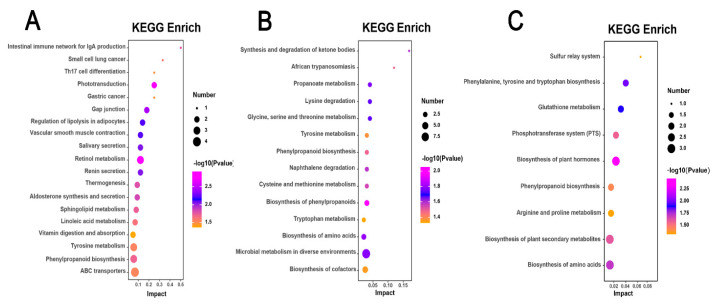
Pathway enrichment analysis of metabolic responses in *H. mutabilis* to *B. tabaci* infestation. Three bubble charts represent KEGG pathway enrichment based on metabolomics data from *H. mutabilis* varieties post *B. tabaci* infestation. (**A**) CBB compared with its four-day post-infestation state (CBB-4d), (**B**) BRHC-4d compared with BRHC, and (**C**) JQS-4d compared with JQS. Each bubble in the charts indicates a specific metabolic pathway, with color showing the significance level (−log10 *p*-value), size reflecting the number of enriched metabolites, and position on the *x*-axis (Impact) illustrating the pathway influence based on topology-analysis-derived impact scores.

Differential metabolites identified in the *H. mutabilis* varieties, specifically the highly susceptible CBB, the moderately resistant BRHC, and the resistant JQS, were mapped to the KEGG database to determine the enriched metabolic pathways following *B. tabaci* infestation. This analysis revealed the involvement of 19, 14, and 9 metabolic pathways, respectively (*p* < 0.05), highlighting significant differences in metabolic responses among these varieties. The differential metabolites in the CBB variety, following *B. tabaci* infestation, were predominantly associated with pathways such as retinol metabolism, phototransduction, gap junction regulation, adipocyte lipolysis, and vascular smooth muscle contraction. The retinol metabolism pathway was particularly significant, with a *p*-value of 0.001195103 and a −log10P of 2.92, and included key metabolites such as retinal, retinoic acid, and beta-carotene (Figure 8A). Differential metabolites of the *B. tabaci*-infested medium-resistant variety BRHC are mainly involved in the biosynthesis of phenylpropanoids, amino acids, microbial metabolism in diverse environments, and propanoate metabolism. The pathway of phenylpropanoid biosynthesis was the most significant (*p* = 0.008822124 and a −log10P of 2.05), with enriched metabolites including sinapoyl aldehyde, 3-(3,4-dihydroxy-5-methoxy)-2-propenoic acid, and 2-hydroxybenzaldehyde (Figure 8B). For JQS, the infestation led to a differential involvement primarily in the biosynthesis of plant hormones, phenylalanine, tyrosine, tryptophan, and other amino acids. The plant hormone biosynthesis pathway was the most highlighted, with a *p*-value of 0.003417717 and a −log10P of 2.47, enriched with metabolites such as S-adenosylmethionine, shikimic acid, and jasmonic acid (Figure 8C). Additionally, post-infestation, the differential metabolites of the highly susceptible and resistant varieties CBB and JQS, respectively, were both involved in the vitamin digestion and phenylpropanoid biosynthesis pathways. Similarly, metabolites from CBB and the moderately resistant BRHC shared involvement in the biosynthesis of phenylpropanoids and tyrosine metabolism pathways. Notably, metabolites from all three varieties (CBB, BRHC, and JQS) were involved in the biosynthesis of phenylpropanoids, underscoring the importance of this pathway in the resistance response to *B. tabaci* infestation. This collective pathway analysis indicates that these pathways are crucial in defining the resistance characteristics of *H. mutabilis* to *B. tabaci*.

## 4. Discussion

### 4.1. Identification and Evaluation of Different H. mutabilis Varieties for Resistance to B. tabaci 

The development of methods to identify resistance to *B. tabaci* in *H. mutabilis* is fundamental, although no unified method or standard has yet been established. Van et al. [31] proposed an evaluation method based on *B. tabaci* egg production and adult and nymph survival rates, but this method is labor-intensive and unsuitable for large-scale applications. Taylor [22] introduced the reproduction index method, validated by Kayani et al. [32] for evaluating cucumber resistance to the southern root-knot nematode, and Millan et al. [23] applied it to analyze and grade tomato resistance to *B. tabaci*. Using these studies as a basis, we adopted the reproduction index method to assess the resistance level of *H. mutabilis* to *B. tabaci*. We found that varieties such as JQS, ZY, and ZFR consistently demonstrated moderate to high resistance over 2 years of evaluations.

Antibiosis and antixenosis affect insect population numbers, which aligns with the goals of IPM to control pest populations; hence, they receive considerable attention [33]. Our investigations revealed significant differences in the antixenosis of different *H. mutabilis* varieties against adult *B. tabaci*, with CBB exhibiting a weaker repelling effect compared to ZY, ZFR, and JQS. Factors influencing plant antixenosis include plant epidermal villi, surface waxes, and volatile compounds [34]. This study focused on the antixenosis of *H. mutabilis* against adult *B. tabaci*, highlighting the need for further research to determine whether surface morphological characteristics correlate with antixenosis. Host plant antibiosis directly affects insect growth, development, and reproduction, serving as a crucial metric for evaluating plant resistance [35]. The variations in the developmental process of *B. tabaci* among *H. mutabilis* varieties observed in this study have been documented in crops such as tomato [23], cucumber [25], pepper, and cassava [36,37]. However, information is limited regarding the developmental processes of *B. tabaci* on different *H. mutabilis* species. We observed significant differences in the developmental calendar period and survival rates of *B. tabaci* among *H. mutabilis* varieties, indicating that these varieties possess antibiosis mechanisms that impede the development and reproduction of *B. tabaci*. The extended larval development period may be due to either a deficiency of essential nutrients in the plant or the presence of secondary metabolites such as proteins, terpenoids, or toxins, which could adversely affect insect behavior or metabolism [38]. Further research into the biochemical components of *H. mutabilis* is necessary to exploit these resistance sources and develop varieties with more stable and long-lasting resistance to *B. tabaci*, providing a scientific basis for the green control of this pest.

### 4.2. Determination of HIPV Components in Different Resistant H. mutabilis Varieties and Analysis of Their Effects on the Olfactory Behavioral Responses of B. tabaci 

Plants deploy various defense mechanisms, including the accumulation of toxic secondary metabolites and the release of volatile signals, to defend against phytophagous insect attacks. Such volatiles play a crucial role in modulating insect orientation, feeding, and aggregation behavior, thereby influencing plant defense responses [39]. We used GC-MS to analyze the major volatile fractions of leaves from different resistant *H. mutabilis* varieties before and after *B. tabaci* infestation. Despite variations in composition and relative content, (+)-camphor consistently ranked the highest. Our findings revealed an increase in volatile species, predominantly alcohols, alkanes, terpenes, ketones, and esters, in both resistant and susceptible *H. mutabilis* after *B. tabaci* infestation. Terpenes, in particular, have garnered attention for their role in plant defense [40]. For instance, beta-caryophyllene and (3E)-4,8-dimethylnona-1,3,7-triene have been shown to attract Nilaparvata lugens [41,42], while (3E,7E)-4,8,12-trimethyltrideca-1,3,7,11-tetraene repels it. These terpenes modulate the behavior of pests and their natural enemies. For example, beta-caryophyllene release from maize roots attracts the natural enemies of the slash-and-burn nightshade moth, reducing root damage [43]. Our study revealed an increased release of specific terpenes, such as (+)-caryophyllene, in highly susceptible varieties of *H. mutabilis*, following *B. tabaci* induction, underscoring the crucial role of terpenes in the defense response of these plants. Furthermore, we identified the release of volatile substances such as l-hexyl-1-octanol and cedrene during infestation by Polyphagotarsonemus latus on tea trees, consistent with our findings, emphasizing the importance of these compounds in indirect plant defense. These results contribute to our understanding of the mechanisms underlying *H. mutabilis* resistance to *B. tabaci* and highlight the potential of using volatile compounds for pest management strategies [44].

### 4.3. Analysis of the Metabolic Response of H. mutabilis Varieties to Infestation by B. tabaci

Pest infestation of plants triggers a cascade of changes in plant metabolites. Regardless of whether a variety is insect-resistant or insect-sensitive, the feeding behavior of insects can induce significant changes in the levels of plant metabolites and expression of related genes [45]. Herein, we performed nontargeted metabolomic analysis on three *H. mutabilis* varieties, highly susceptible, moderately resistant, and insect-resistant, before and after *B. tabaci* infestation. This revealed complex differential metabolite responses, with the susceptible varieties showing more extensive and drastic metabolic changes induced by *B. tabaci*, which is consistent with findings reported by Tu [45]. 

Flavonoids are crucial in the plant response to insect stress, inhibiting insect feeding, development, and oviposition and acting as signaling molecules to enhance the defense against pathogens [46]. Yao et al. [47] found that high levels of flavonoids in tomato effectively impeded *B. tabaci* feeding and egg-laying and revealed that these compounds significantly reduced the stinging and bast-feeding efficiency of *B. tabaci*, as shown by prickling potential mapping, thus reducing the primary and secondary transmission of tomato yellow leaf curl virus. Li et al. [48] found that flavonoid metabolism positively regulates the resistance of Zanthoxylum bungeanum. In addition to flavonoids, ascorbic acid has an important role in the plant defense system. As an important component of the antioxidant system, ascorbic acid reduces damage to photosynthesis sites in chloroplasts, improves the photosynthetic capacity, and slows plant aging [49]. When plants face insect infestation, terpenoids play a role in defense by either directly repelling the insects or indirectly attracting their natural enemies. For example, limonene-1,2-diol can effectively repel whiteflies in celery [50]. In plant antipathology, 3-indoleacetonitrile has been used to synthesize derivatives with significant inhibitory activity against pathogens such as Alternaria alternata and Botrytis cinerea [51]. All these metabolites commonly associated with plant disease resistance were detected in this study plus another 15 metabolites characterized by resistance, such as 6-hydroxynicotinic acid, 5-methylbarbituric acid, (3S)-3,6-diaminohexanoate, D-lycopyrrolate, and glutamine-based alanine. These specific metabolites have potential application value in many areas, can provide an important reference for the selection of high-quality *B. tabaci*-resistant germplasm resources, and are expected to be developed as biomarkers for resistance breeding.

Plant phenylpropanoid metabolism is one of the key pathways for the formation of disease resistance defenses in plants, producing various resistance-related substances such as phenols, flavonoids, and lignans. The pathway passes through branch acids, prenylates, and transamination to form phenylalanine, which consequently generates cinnamaldehyde catalyzed by phenylalanine ammonia-lyase, among others, followed by the cleavage of cinnamic acid to coumaric acid by cinnamate 4-hydroxylase (C_4_H) [52]. Herein, the phenylpropanoid metabolic pathway was significantly enriched across different comparison groups, indicating its crucial role in plant disease resistance and insect defense. Using KEGG metabolic pathway analysis, we analyzed the differential metabolites in the same metabolic pathway induced by *B. tabaci* in different resistant varieties of *H. mutabilis* and found that the differential metabolites differed among resistant varieties of *H. mutabilis*. Insect-resistant varieties accumulated more methyl eugenol (downregulated), isoeugenol (downregulated), sinapaldehyde (upregulated), and 3-(3,4-dihydroxy-5-methoxy-N-acetylserotonin)-2-propenoic acid (downregulated). Conversely, the susceptible varieties accumulated more 4-hydroxycinnamic acid (upregulated), 7-hydroxycoumarin (upregulated), and methyl eugenol (downregulated). 4-Hydroxycinnamic acid and sinapaldehyde are involved in the synthesis of S-type lignin monomers, indicating that *H. mutabilis* reinforces its physical barriers by synthesizing lignin structural components to resist feeding by *B. tabaci*. Numerous studies have reported a significant relationship between lignin and plant resistance to insects and diseases [53]. The synthesis of lignin not only strengthens the physical structure of the plant but also plays a critical role in defense mechanisms against various biotic stresses. This enhancement of physical barriers limits the ability of pests such as *B. tabaci* to penetrate and damage plant tissues, effectively reducing the effect of their attacks. Coumarins are aromatic polyphenolic substances that help protect plants against diseases and pests, safeguarding them from fungal infections [54]. Our research found that after whiteflies fed on susceptible *H. mutabilis* varieties, the content of coumarin compounds significantly increased. This suggests that susceptible varieties also activate their own defense systems upon insect attack and that coumarin compounds may play an important role in the insect resistance response of *H. mutabilis*. The study of such metabolites in *H. mutabilis* helps to provide guidance for breeding resistant lines.

The in-depth study of these key metabolites provides a scientific basis for understanding the defense mechanism of *H. mutabilis* and may offer important guidance for breeding new insect-resistant varieties. Subsequent research should focus on gene function validation through joint multiomics analysis to clarify the resistance mechanisms of *H. mutabilis* to *B. tabaci*.

## Figures and Tables

**Table 1:** The known *H. mutabilis* leaf volatiles identified by GC-MS.

Chemical Class	Compound	Rt (min)	CAS#	Formula
Alkanes	Dodecane	17.780	112−40−3	C_12_H_26_
	Heptadecane	19.895	629−78−7	C_17_H_36_
	Hexadecane	21.210	544−76−3	C_16_H_34_
	4-Methyltetradecane	22.19	25,117−24−2	C_15_H_32_
	10-Methyleicosane	22.35	54,833−23−7	C_21_H_44_
	2,6,10-Trimethyldodecane	24.835	3891−98−3	C_15_H_32_
	5-Methyltetradecane	22.05	25,117−32−2	C_15_H_32_
	Tetradecane	23.31	629−59−4	C_14_H_30_
	Pentadecane	25.835	629−62−9	C_15_H_32_
	Nonadecane	28.135	629−92−5	C_19_H_40_
	Phytane	21.54	638−36−8	C_20_H_42_
	Eicosane	27.355	112−95−8	C_20_H_42_
Alcohols	DL-Menthol	17.255	15,356−70−4	C_10_H_20_O
	2-Butyl-1-octanol	24.560	3913−02−8	C_12_H_26_O
	2-Ethylhexanol	12.56	104−76−7	C_8_H_18_O
	Borneol	16.995	507−70−0	C_10_H_18_O
	Phytol	21.35	150−86−7	C_20_H_40_O
	Cedrol	28.470	77−53−2	C_15_H_26_O
	Hexyl decanol	29.34	2425−77−6	C_16_H_34_O
	6-Methylheptanol	15.84	1653−40−3	C8H_18_O
	2-Decen-1-ol	16.910	18,409−18−2	C_10_H_20_O
Terpenes	Neophytadiene	31.785	504−96−1	C_20_H_38_
	(+)-Limonene	12.4	5989−27−5	C_10_H_16_
	(+)-Longifolene	22.615	1137−12−8	C_15_H_24_
	Caryophyllene	23.545	475−20−7	C_15_H_24_
	Valencene	23.545	4630−07−3	C_15_H_24_
	α-Cedrene	23.715	469−61−4	C_15_H_24_
	(+)-Cuparene	26.04	16,982−00−6	C_15_H_22_
Benzene derivatives	Naphthalene	17.445	91−20−3	C_10_H_8_
	1,2,4,5-Tetramethylbenzene	15.315	95−93−2	C_10_H_14_
Aldehydes	Lauryl aldehyde	16.930	112−54−9	C_12_H_24_O
	Capric aldehyde	17.985	112−31−2	C_10_H_20_O
Ketones	2-Methyl-5-(1-methylethenyl) cyclohexanone	14.950	7764−50−3	C_10_H_16_O
	(+)-camphor	16.295	464−49−3	C_10_H_16_O
	2-Undecanone	20.450	112−12−9	C_11_H_22_O
Esters	Octyl acrylate	18.49	103−11−7	C_11_H_20_O_2_
	2,2,4-Trimethyl-1,3-pentanediol diisobutyrate	27.87	6846−50−0	C_16_H_30_O_4_
	Linalyl acetate	36.505	115−95−7	C_12_H_20_O_2_
Acids	Stearic acid	35.42	57−11−4	C_18_H_36_O_2_
	Palmitic acid	33.395	57−10−3	C_16_H_32_O_2_

Rt (min) represents the retention time, CAS# indicates the CAS number.

**Table 2:** Screening for differential metabolites for resistance characterization.

Compound	Mzmed	Rtmed	Fold-Change	Log2FC	*p* Value	VIP	Regulated
Catechol	111.0918	33	0.5	−1.01	0.0292	2.0928	down
Isochavicol	134.0812	99	0.51	−0.98	0.0311	1.9758	down
6-Hydroxynicotinic acid	139.123	749.3	3.8	1.93	0.0437	2.0028	up
5-Methylbarbiturate	142.035	79.7	0.47	−1.09	0.0265	1.9594	down
(3S)-3,6-Diaminohexanoate	147.1168	359.6	2.47	1.3	0.0202	2.1068	up
D-Lyxose	151.0346	665.7	0.44	−1.19	0.0471	1.9746	down
3-Indoleacetonitrile	157.085	39.1	0.32	−1.66	0.0134	2.1457	down
Limonene-1,2-diol	171.138	362.6	1.58	0.66	0.0340	2.0571	up
Ascorbate	176.972	46.1	0.94	−0.09	0.0407	1.8343	down
trans-Isoasarone	191.1076	491	0.52	−0.93	0.0331	1.8886	down
gamma-Glutamylalanine	219.0974	103.3	0.46	−1.11	0.0319	1.9997	down
L-Arogenate	228.0843	152.9	2.67	1.42	0.0248	2.0473	up
Zeranol	323.1853	690	0.52	−0.93	0.0269	2.0429	down
L-Norvaline	115.9189	871	1.45	0.54	0.0111	2.5431	up
Guanidoacetic acid	116.9266	889.6	1.59	0.67	0.0376	2.5345	up

Mzmed: Median *m*/*z* (mass-to-charge ratio) in mass spectrometry. Rtmed: Median retention time in chromatography. Fold-change: Expression level ratio between conditions. Log2FC: Logarithm (base 2) of the fold-change. VIP: Variable importance in projection in discriminant analysis.

## Data Availability

The data that support the findings of this study are available from the corresponding author upon reasonable request. Researchers interested in accessing the data should contact the lead author via email at 18352887796@163.com.

## Supplementary Materials

The following supporting information can be downloaded at https://www.mdpi.com/article/10.3390/insects15060454/s1, Figure S1: Volatile collection device; Table S1: Information on 8 volatile compounds used in olfactory behavioral response tests; Table S2: Field resistance evaluation of 14 *H. mutabilis* cultivars to *B. tabaci* (2021); Table S3: Field resistance evaluation of 14 *H. mutabilis* cultivars to *B. tabaci* (2022); Table S4: Development periods of *B. tabaci* on different *H. mutabilis* varieties; Table S5: Survival rate of *B. tabaci* on different *H. mutabilis* varieties; Table S6: Behavioral responses of *B. tabaci* to different concentrations of standard compounds; Table S7: Screening for metabolites with differential susceptibility characteristics.
