# Supplementary material for: Identification and Characterization of Hibiscus mutabilis Varieties Resistant to Bemisia tabaci and Their Resistance Mechanisms
Source: Insects. 2024 Jun 14;15(6):454. doi: 10.3390/insects15060454 (PMC11203673; doi:10.3390/insects15060454)
Supplement: Supplementary file 1 [file insects-15-00454-s001.zip › insects-3030630-supplementary.pdf]

Supplementary Materials:

Figure S1. Closed-loop system for collecting plant volatile compounds. This schematic represents the setup of a closed-loop volatile collection system designed for capturing volatile organic compounds from *H. mutabilis* leaves. The system encloses leaves in a specialized collection bag, ensuring a sealed environment. VOCs emitted by the leaves are drawn through an odorless conduit by an air pump, passing first through a flowmeter to monitor the rate, and then through activated charcoal to capture the volatiles. The gas flow is maintained at a constant rate of 0.5 L/min for 60 minutes at an ambient temperature of 25°C within an odor-free chamber.

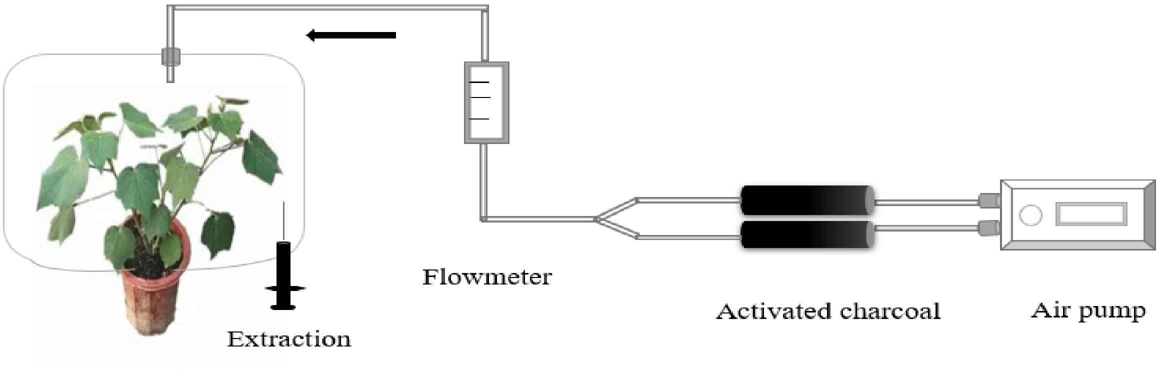

Table S1. Information on 8 volatile compounds used in olfactory behavioral response tests.

Reagent	CAS Number	Purity	Source
Eicosane	112-95-8	GC⩾99.5%	Aladdin
Dodecyl aldehyde	112-54-9	GC⩾95.0%	Aladdin
Linalyl acetate	115-95-7	GC⩾96.0%	Macklin
Stearic acid	57-11-4	GC⩾99.0%	Macklin
Cedrol	77-53-2	GC⩾98.0%	Aladdin
2-Ethylhexanol	104-76-7	GC⩾99.5%	Macklin
2-butyl-1-octanol	3913-02-8	GC⩾98.0%	Aladdin
Alpha-Cedrene	469-61-4	GC⩾95.0%	Aladdin

Table S2. Field Resistance Evaluation of 14 *H. mutabilis* Cultivars to *B. tabaci* (2021).

Variety	Adult numbers (head·leaf^{-1})			Mean (head·leaf^{-1})	Egg numbers (grain·leaf^{-1})	Reproduction index	Degree of resistance
	24 h	48 h	72 h				
CBH	3.13 ± 0.60 cde	2.80 ± 0.62 bcd	3.00 ± 0.60 bcd	2.98 ± 0.10 e	7.13 ± 1.84 ef	23.83 ± 6.15 ef	MR
ZFR	1.60 ± 0.39 e	1.60 ± 0.59 d	1.33 ± 0.44 e	1.51 ± 0.09 f	5.07 ± 1.27 f	16.93 ± 4.23 f	R
JQS	2.33 ± 0.55 e	2.13 ± 0.91 cd	1.93 ± 0.66 cde	2.13 ± 0.12 f	7.13 ±1.42 ef	23.83 ± 4.74 ef	MR
QY	3.33 ± 0.74 bcde	3.20 ± 0.85 bcd	3.87 ± 1.36 ab	3.47 ± 0.21 cde	18.00 ± 2.88 bc	60.13 ± 9.61 bc	S
ZH	3.33 ± 0.72 bcde	3.07 ± 0.57 bcd	2.73 ± 0.39 bcde	3.04 ± 0.17 e	19.67 ± 4.45 bc	65.70 ± 14.87 bc	S
BRHC	4.40 ± 0.80 abcd	3.93 ± 1.09 abc	3.67 ± 1.35 b	4.00 ± 0.21 cd	11.93 ± 2.84 cdef	39.87 ± 9.48 cdef	MR
CX	2.93 ± 0.78 de	3.13 ± 0.91 bcd	3.47 ± 1.19 bc	3.18 ± 0.16 de	17.60 ± 3.21 bc	58.80 ± 10.73 bc	S
MDH	4.13 ± 0.73 abcd	4.07 ± 0.94 abc	2.47 ± 0.60 bcde	3.56 ± 0.54 cde	16.67 ± 3.57 cde	55.68 ± 11.92 cde	S
YR	3.40 ± 0.74 bcde	3.33 ± 0.46 bcd	2.93 ± 0.54 bcde	3.22 ± 0.15 de	26.87 ± 4.55 ab	89.76 ± 15.21 ab	S
MDF	4.87 ± 1.23 abc	4.60 ± 1.68 ab	3.40 ± 1.05 bc	4.29 ± 0.45 bc	7.73 ± 1.54 def	25.84 ± 5.16 def	MR
JBY	3.00 ± 0.51 de	3.07 ± 0.81 bcd	3.27 ± 0.56 bc	3.11 ± 0.08 e	9.80 ± 1.76 cdef	32.74 ± 5.90 cdef	MR
ZY	2.20 ± 0.17 e	2.27 ± 0.32 cd	1.60 ± 0.24 de	2.02 ± 0.21 f	4.00 ± 0.41 f	13.36 ± 1.36 f	R
JXZ	5.07 ± 1.33 ab	5.40 ± 1.81 a	5.33 ± 1.63 a	5.27 ± 0.10 a	17.13 ± 4.71 cd	57.24 ± 15.74 cd	S
CBB	5.73 ± 0.93 a	5.40 ± 0.84 a	4.07 ± 0.69 ab	5.07 ± 0.51ab	29.93 ± 4.06 a	100.00 ± 13.56 a	HS

Note: The data in the table is Mean ± S.E. Different letters above the same column indicate significant difference between treatment at 0.05 level.

Resistance level: HR=high resistance; R=resistant; MR=moderate resistance; S=sense; HS=high sense.

Table S3. Field Resistance Evaluation of 14 *H. mutabilis* Cultivars to *B. tabaci* (2022).

Variety	Adult numbers (head·leaf^{-1})			Mean (head·leaf^{-1})	Egg numbers （grain·leaf^{-1}）	Reproduction index	Degree of resistance
	24 h	48 h	72 h				
CBH	6.33 ± 1.74 abcde	5.33 ± 0.97 ab	3.67 ± 1.08 ab	5.11 ± 0.78 ab	17.60 ± 4.33 ab	81.99 ± 20.19 ab	S
ZFR	3.07 ± 0.84 e	2.20 ± 0.48 cd	1.07 ± 0.55 cde	2.11 ± 0.58 b	8.27 ± 2.00 bc	38.51 ± 9.34 bc	MR
JQS	4.40 ± 0.73 cde	1.53 ± 0.40 d	0.47 ± 0.47 e	2.13 ± 1.17 b	4.13 ± 1.16 c	19.25 ± 5.41 c	R
QY	7.07 ± 2.23 abcd	3.67 ± 0.94 bcd	2.93 ± 0.93 abcd	4.56 ± 1.27 ab	14.53 ± 4.05 abc	67.70 ± 18.85 abc	S
ZH	3.60 ± 1.72 de	1.00 ± 0.38 d	2.33 ± 0.82 bcde	2.31 ± 0.75 b	7.07 ± 1.92 bc	32.92 ± 8.94 bc	MR
BRHC	3.27 ± 1.30 e	2.53 ± 1.06 cd	1.93 ± 0.76 bcde	2.58 ± 0.39 ab	7.87 ± 2.51 bc	36.65 ± 11.69 bc	MR
CX	4.07 ± 0.71 de	3.20 ± 1.30 bcd	2.53 ± 0.83 bcde	3.2 7± 0.45 ab	9.93 ± 2.86 bc	46.27 ± 13.33 bc	MR
MDH	6.60 ± 2.37 abcde	3.47 ± 0.50 bcd	1.67 ± 0.69 bcde	3.91 ± 1.44 ab	6.47 ± 1.57 c	30.12 ± 7.29 c	MR
YR	8.53 ± 2.77 ab	4.47 ± 0.99 abc	2.07 ± 1.07 bcde	5.02 ± 1.89 ab	10.60 ± 4.06 bc	49.38 ± 18.91 bc	MR
MDF	9.73 ± 3.12 a	4.73 ± 0.83 abc	1.07 ± 0.39 cde	5.18 ± 2.51 ab	8.60 ± 2.71 bc	40.06 ± 12.63 bc	MR
JBY	7.80 ± 1.95 abc	3.07 ± 0.65 bcd	1.47 ± 0.66 bcde	4.11 ± 1.90 ab	8.80 ± 0.83 bc	40.99 ± 3.85 bc	MR
ZY	50.00 ± 2.00 bcde	2.80 ± 1.07 bcd	0.67 ± 0.43 de	2.82 ± 1.25 ab	6.07 ± 1.78 c	28.26 ± 8.28 c	MR
JXZ	4.00 ± 0.67 de	2.27 ± 0.56 cd	3.13 ± 0.51 abc	3.13 ± 0.50 ab	11.20 ± 4.58 bc	52.17 ± 21.35 bc	S
CBB	8.40 ± 1.83 ab	6.93 ± 1.24 a	4.87 ± 1.25 a	6.73 ± 1.02 a	21.47 ± 5.90 a	100.00 ± 27.50 a	HS

Note: The data in the table is Mean ± S.E. Different letters above the same column indicate significant difference between treatment at 0.05 level.

Resistance level: HR=high resistance; R=resistant; MR=moderate resistance; S=sense; HS=high sense.

Table S4. Development periods of *B. tabaci* on different *H. mutabilis* varieties.

Variety	Developments of *B.tabaci* of different stages（d）					
	Egg	1st instar nymph	2nd instar nymph	3rd instar nymph	Pseudopupa	Egg-pseudopupa
CBB	9.19 ± 0.41 b	6.32 ± 0.62 a	4.43 ± 0.60 b	3.63 ± 0.03 a	4.31 ± 0.52 b	27.89 ± 1.70 b
ZFR	10.17 ± 0.07 ab	5.50 ± 0.68 a	4.71 ± 0.86 ab	5.40 ± 0.94 a	6.26 ± 0.14 ab	32.04 ± 0.85 a
JQS	10.52 ± 0.27 a	6.44 ± 0.45 a	3.32 ± 0.50 b	4.25 ± 0.55 a	6.61 ± 0.79 ab	31.14 ± 0.33 ab
BRHC	9.91 ± 0.68 ab	7.05 ± 0.35 a	3.63 ± 0.49 b	4.48 ± 0.43 a	6.92 ± 0.61 a	31.99 ± 1.33 a
MDH	10.71 ± 0.37 a	6.08 ± 0.57 a	3.63 ± 0.43 b	3.78 ± 0.71 a	5.67 ± 1.40 ab	29.87 ± 1.46 ab
ZY	9.08 ± 0.40 b	6.61 ± 0.49 a	6.26 ± 0.32 a	4.50 ± 1.20 a	7.30 ± 0.06 a	33.75 ± 1.19 a
JXZ	9.58 ± 0.19 ab	5.31 ± 0.75 a	4.29 ± 0.31 b	3.71 ± 0.29 a	4.95 ± 0.48 ab	27.85 ± 0.85 b

Note: The data in the table is Mean ± S.E. Different letters above the same column indicate significant difference between treatment at 0.05 level.

Table S5. Survival rate of *B. tabaci* on different *H. mutabilis* varieties.

Variety	Survival rate of different stages（%）					
	Egg	1st instar nymph	2nd instar nymph	3rd instar nymph	Pseudopupa	Egg - pseudopupa
CBB	88.06 ± 1.00 a	87.74 ± 2.29 a	91.55 ± 0.12 a	90.69 ± 2.81 a	91.64 ± 1.41 a	62.08 ± 3.07 a
ZFR	86.67 ± 5.09 a	89.96 ± 1.88 a	90.07 ± 1.04 a	84.47 ± 8.28 a	88.52 ± 0.98 a	52.22 ± 4.84 ab
JQS	83.68 ± 2.68 a	83.15 ± 4.08 a	88.65 ± 3.30 ab	87.35 ± 2.11 a	85.21 ± 3.27 a	46.43 ± 2.06 b
BRHC	88.96 ± 1.02 a	89.34 ± 3.17 a	79.45 ± 7.03 b	83.57 ± 4.46 a	83.33 ± 8.48 a	46.02 ± 10.90 b
MDH	83.94 ± 2.42 a	88.84 ± 3.27 a	91.88 ± 0.72 a	91.15 ± 0.86 a	95.01 ± 2.66 a	59.22 ± 2.15 a
ZY	86.96 ± 1.68 a	85.19 ± 7.99 a	90.72 ± 1.82 a	91.41 ± 1.62 a	84.86 ± 2.64 a	51.99 ± 4.82 ab
JXZ	87.92 ± 1.05 a	88.70 ± 2.30 a	90.01 ± 1.83 a	92.32 ± 1.22 a	89.82 ± 3.19 a	59.35 ± 3.90 a

Note: The data in the table is Mean ± S.E. Different letters above the same column indicate significant difference between treatment at 0.05 level.

Table S6. Behavioral responses of *B. tabaci* to different concentrations of standard compounds.

Compound	Reaction rate	Mass concentration (mg/mL)	Select response rate	Selectivity coefficient
Eicosane	82.53 ± 0.86	CK	22.40 ± 1.13 a	—
		10-1	26.10 ± 1.54 a	0.0370 ± 0.0124
		10-2	26.00 ± 1.73 a	0.0360 ± 0.0254
		10-3	25.49 ± 1.49 a	0.0308 ± 0.0245
Dodecanal	69.44 ± 4.22	CK	15.04 ± 3.37 b	—
		10-1	24.74 ± 2.12 ab	0.097 ± 0.0406
		10-2	32.27 ± 6.25 a	0.1723 ± 0.0898
		10-3	27.95 ± 2.95 a	0.1291 ± 0.0342
Linalyl acetate	74.71 ± 5.20	CK	21.06 ± 2.45 a	—
		10-1	23.81 ± 5.98 a	0.0275 ± 0.075
		10-2	24.46 ± 4.06 a	0.0341 ± 0.0616
		10-3	30.67 ± 5.26 a	0.0961 ± 0.0396
Stearic acid	82.00 ± 2.01	CK	42.18 ± 2.24 a	—
		10-1	15.66 ± 2.77 c	-0.2653 ± 0.0415
		10-2	17.91 ± 2.90 bc	-0.2427 ± 0.0501
		10-3	24.25 ± 2.00 b	-0.1793 ± 0.017
Cedar alcohol	83.79 ± 2.44	CK	15.41 ± 2.11 b	—
		10-1	34.35 ± 4.00 a	0.1894 ± 0.0521
		10-2	24.08 ± 4.07 ab	0.0867 ± 0.0307
		10-3	26.17 ± 3.71 ab	0.1077 ± 0.0566
2-Ethylhexanol	77.24±2.88	CK	32.49 ± 1.44 a	—
		10-1	13.94 ± 0.66 b	-0.1962 ±0.010
		10-2	28.73 ± 3.80 a	-0.0505 ± 0.0385
		10-3	24.84 ± 3.10 a	-0.0881 ± 0.031
2-Butyl-1-octanol	77.52 ± 1.75	CK	33.88 ± 0.85 a	—
		10-1	17.16 ± 1.13 c	-0.1672 ± 0.0185
		10-2	21.38 ± 2.22 c	-0.125 ± 0.0299
		10-3	27.57 ± 2.27 b	-0.0631 ± 0.0161
α-Cedrene	78.02 ± 1.40	CK	18.89 ± 1.73 b	—
		10-1	24.54 ± 3.14 ab	0.0564 ± 0.0312
		10-2	31.43 ± 4.80 a	0.1253 ± 0.0594
		10-3	25.14 ± 1.36 ab	0.0625 ± 0.0217

Note: Different lowercase letters of the same data under the same compound indicate significant difference（$p < 0.05$）.

Table S7. Screening for metabolites with differential susceptibility characteristics.

Compound	mzmed	rtmed	Fold-change	Log2FC	P value	VIP	Regulated
Betaine	118.0858	94.8	1.89	0.92	0.0473	1.7833	up
Gamma-terpinene	137.1322	987	0.55	-0.86	0.0080	2.0861	down
Indoleacetaldehyde	159.0653	577.7	2.78	1.47	0.0360	1.7279	up
4-Hydroxycinnamoylmethane	162.0762	134.9	1.46	0.55	0.0058	2.1029	up
m-Coumaric acid	165.0537	356.3	0.72	-0.47	0.0071	2.0831	down
2-Oxo-4-phenylbutyric	179.0705	516.2	1.81	0.85	0.0395	1.8602	up
N-Acetylglutamic acid	190.0711	213.9	0.49	-1.02	0.0221	1.8571	down
3-Methyl-L-tyrosine	195.1009	562	2.14	1.1	0.0428	1.6778	up
N-Acetylserotonin	218.2112	493.8	1.25	0.32	0.0031	2.2229	up
D-Lysopine	219.1338	148.7	1.25	0.32	0.0179	1.9382	up
Cerulenin	224.1282	252.9	1.53	0.61	0.0427	1.6929	up
Hydroxykynurenine	224.1281	316.7	0.49	-1.02	0.0207	1.8669	down
(S)-Abscisic acid	247.1328	402.8	1.88	0.91	0.0462	1.7982	up
2'-Deoxyadenosine	252.1092	244.9	1.71	0.78	0.0276	1.8075	up
D-Allulose 6-phosphate	261.0577	149.3	4.47	2.16	0.0014	2.3372	up
Androstanedione	289.216	780.7	0.71	-0.49	0.0076	2.0871	down
Exemestane	296.1699	248.2	0.32	-1.64	0.0117	2.0072	down
Phytosphingosine	318.2999	705.6	0.38	-1.41	0.0487	1.7774	down
18-Hydroxycorticosterone	363.2148	676.1	2.35	1.23	0.0202	1.9094	up
Peregrinol diphosphate	469.2033	604.4	1.67	0.74	0.0474	1.6702	Up
Canthaxanthin	565.4041	851.8	0.56	-0.84	0.0258	1.9983	down
Tribuloside	595.1414	533.3	0.47	-1.07	0.0307	1.7790	down
Phenyl acetate	134.8934	873.2	0.5	-1.01	0.0010	2.2862	down
4-Hydroxycinnamic acid	163.0396	232.2	2.9	1.53	0.0023	2.2590	up
Norepinephrine	168.0649	248.5	2.23	1.16	0.0208	1.9351	up
4-Quinolinecarboxylic acid	173.117	475.3	2.15	1.1	0.0035	2.1206	up
3,4-Dihydroxymandelic acid	183.0289	400.9	0.76	-0.39	0.0150	1.8439	down
4-Hydroxycinnamic acid	163.0396	232.2	2.9	1.53	0.0023	2.2590	up
Citrinin	249.0768	428.6	1.2	0.27	0.0172	1.9633	up
Stearic acid	283.2637	924.8	0.39	-1.34	0.0066	1.9948	down
all-trans-Retinoic acid	299.2586	902.3	1.61	0.68	0.0268	1.8828	up
9,10-12,13-Diepoxyoctadecanoate	312.2254	721.5	3.32	1.73	0.0337	1.7268	up
Cyclic GMP	344.0393	229.4	0.43	-1.22	0.0245	1.7620	down
Enalaprilat	347.1712	485.4	2.05	1.03	0.0268	1.6442	up
